# Children at play: The role of novices in the production of Europe’s earliest Upper Paleolithic ceramics

**DOI:** 10.1371/journal.pone.0309107

**Published:** 2024-10-25

**Authors:** Rebecca Farbstein, April Nowell

**Affiliations:** 1 Independent Scholar, London, United Kingdom; 2 Department of Anthropology, University of Victoria, Victoria, British Columbia, Canada; Universita degli Studi di Ferrara, ITALY

## Abstract

Although archaeologists are learning more about the lives of Upper Paleolithic children, the significant contributions they made to the welfare of their communities, including their role in craft production, remain understudied. In the present study, we use high resolution photographs of 489 ceramic artifacts from Dolní Věstonice I and II, Pavlov I and VI, and Předmostí, five archaeological sites in Czechia (ca. 30,000 BP) to address two questions: 1. Can the ceramic products of novices be distinguished from those made by experts? 2. If so, can we tell if these novices were children? To address these questions, we documented variables known ethnographically and archaeologically to be associated with learners in a sample from these five sites. The sample is composed of fired (“ceramic”) and unfired (“sedimentary”) anthropomorphic and zoomorphic figurines, non-diagnostic figurine fragments and a sample of the so-called "pellets" from one site, Pavlov I. Our results support the hypothesis that ceramic objects are the products of novices, and in many cases, these novices are children. Our findings have implications for inter-generational knowledge transmission, the role of children in craft production and the importance of learning through play.

## Introduction

Increasing attention to the archaeological study of children and adolescents in the European Upper Paleolithic record [1–15] has led to a greater understanding of the richness of the lives they led. For example, footprints [16] and handprints [17], provide direct evidence of their participation in the production of cave art, as well as the games they played and their relationships with others in their communities. Rare examples of engraved and painted images of children [2] help to presence them while figurines of children and adolescents provide data on clothing [18]. Bioarchaeological studies of burials offer further direct evidence of children in terms of health, morbidity and diet as well as mortuary practices, clothing, personal ornaments, and objects of meaning to the children and their communities in the form of grave goods [8 and references therein]. Archaeologists have also uncovered indirect evidence of children’s toys [8, 12, 18] and of children’s participation in communities of practice centered around stone tool manufacture [1, 8, 19–23].

There are, however, significant gaps in our knowledge of the lives of Paleolithic children [10]. One of the areas that remains understudied is children’s contribution to craft production. One of the reasons for this gap may be the assumptions Western archaeologists bring to a study of children and childhood in the past. Kamp [24, p.18] argues that archaeologists often assume that children are “incapable of performing complex tasks or assuming responsibility.” Similarly, Kohut [25, p.153] observes that “Western notions of childhood as an innocent, carefree period…may have hindered alternative views of the experience of childhood throughout space and time.” It is clear, however, from ethnographic and archaeological data that children around the world, and across time, have commonly been involved in tasks including herding, fetching water, harvesting vegetables, running market stalls, collecting firewood, tending animals, cleaning and sweeping, working as musicians, serving as soldiers, and caring for younger siblings [8, 24]. Furthermore, in many societies, children were actively engaged in the production of textiles, ceramics, stone tools and beads among other craft items [8, 25–30]. These activities are often discernible in the archaeological record if we know where to look and this undertaking begins with asking questions about children as makers in the past.

Accordingly, in this paper, we use high resolution photographs of ceramic artifacts from Dolní Věstonice I (DV I), Dolní Věstonice II (DV II), Pavlov I, Pavlov VI, and Předmostí, five archaeological sites in eastern Czechia dating to ca. 30,000 BP, to address two questions: 1. Can the ceramic products of novices be distinguished from those made by experts? 2. If so, can we discern if these novices were children? Novices are individuals who are new or inexperienced in a field or situation. Not all novices are children as it is possible to learn a new skill at any age. Similarly, not all children are novices as some quickly acquire expertise [24]. Nonetheless, in the Paleolithic, it is reasonable to assume that while not everyone might learn to make ceramics, those who did began at an early age [8]. Our study sample includes both fired and unfired anthropomorphic and zoomorphic clay figurines, figurine fragments and non-diagnostic pellets, all of which were made from a local, loess-rich sedimentary paste. To explore whether these artifacts were made by novices, we documented variables known ethnographically and archaeologically to be associated with learners and compared them to the same variables recorded in art objects made from ivory, bone and antler from these same sites. Our results support the hypothesis that ceramic objects are the products of novices and in many cases, these novices are children. By way of discussion and conclusion, we consider the implications of our findings for inter-generational knowledge transmission, the role of children in craft production and the importance of learning through play.

## Methods and materials

### Variables associated with novice makers

In this study, we drew upon variables known ethnographically and archaeologically to be associated with novice makers, with a particular focus on children and adolescents learning to make ceramics and stone tools. However, not all variables were applicable to our sample. For example, while many studies focus on the quality and nature of painted designs on pots [e.g., 27, 31–33], none of the ceramic artifacts from the Upper Paleolithic preserve any evidence of being painted. Similarly, novices are often associated with poorer quality raw materials, particularly where these materials are scarce [34]. The Paleolithic ceramic artifacts from our sample were all made from the same basic sediment, although it is possible to argue that the local, loess-rich sediment that was used to make Pavlovian ceramics was more abundant and thus perhaps a less desired or ‘lower quality’ raw material than the ivory, bone and antler used to make other symbolic material culture at the same sites. Accordingly, we focus on five key variables that were most recoverable in the assemblages of Paleolithic art considered in this paper:

#### Size

Smaller sized artifacts are likely to have been produced by novices, while experts are more likely to produce larger artifacts [24, 32, 35–43]. While small artifact size can be the result of a number of variables (e.g., function, raw material availability or level of group mobility; see Discussion in [8]), Kaaronen et al. [44] demonstrate the cross-cultural importance of body-based measurements in ergonomic design; they argue that size is a significant factor in the design of objects such as tools, weapons, clothing and footwear and can be directly linked to the user’s body proportions.

#### Asymmetry

Novices produce asymmetrical ceramic artifacts more often than experts [24, 40, 45]. We prefer this category to ‘irregular’ or ‘crudely made’, as novice produced ceramics are sometimes described [e.g., 38] because it is more quantifiable and less subjective.

#### Complexity of chaînes opératoires

Although chaîne opératoire has historically often been used to document consistency in the production of mainly functional material culture within a cultural group, it also has the potential to be used to uncover variability and diversity. Novices are associated with less complex production sequences or chaînes opératoires [5, 24, see also 46] and simpler techniques such as pinching, pressing and pulling rather than coiling in the case of ceramics [24, 40, 45, 47]. We can also compare the relative complexity of chaînes opératoires across different classes of raw material (e.g., ceramic vs. ivory, or ceramic vs. bone) to gain insight if novices worked with some materials more often than others.

#### Experimentation and heterogeneity

Novices produce artifacts which reveal higher degrees of experimentation and heterogeneity in both production and final form/appearance [27; see also 1]. Köhler [40, p.129], who studied how Sangopari (Côte d’Ivoire) novices learn to make pottery, writes “…girls who are by themselves are more likely to try out different techniques than when they are together with a competent woman, as she may regularly intervene and correct the learners.” This variable can be contrasted with evidence for seriation (i.e., the production of standardized objects) and homogeneity, as these characteristics may, by extension, offer evidence of forethought and planning, and suggest a more experienced, skillful craftsperson at work.

#### Non-productivity

When objects were made without a focus on a long use-life after production, and when they were found *in situ* at the location where they were made, they are considered to have been made ‘non-productively’ [24]. The practice of making the object itself, and the play (or playful experimentation) involved in production, are the primary motivations for making such artifacts [see also 48–50]. These non-productive pieces were more likely to have been made by novices and/or children [see also 21, 22, 24, 28, 51, 52]. Köhler [40, p.128] watching three children learning to make pots observes, “nothing the learners produced survived the end of this session, as the children were either unable to construct a pot, or the pots they made were destroyed afterwards. In addition, the smallest child added so much water to her own piece of clay and to the other pieces that they all fell apart.”

### Sites

**Fig 1:**
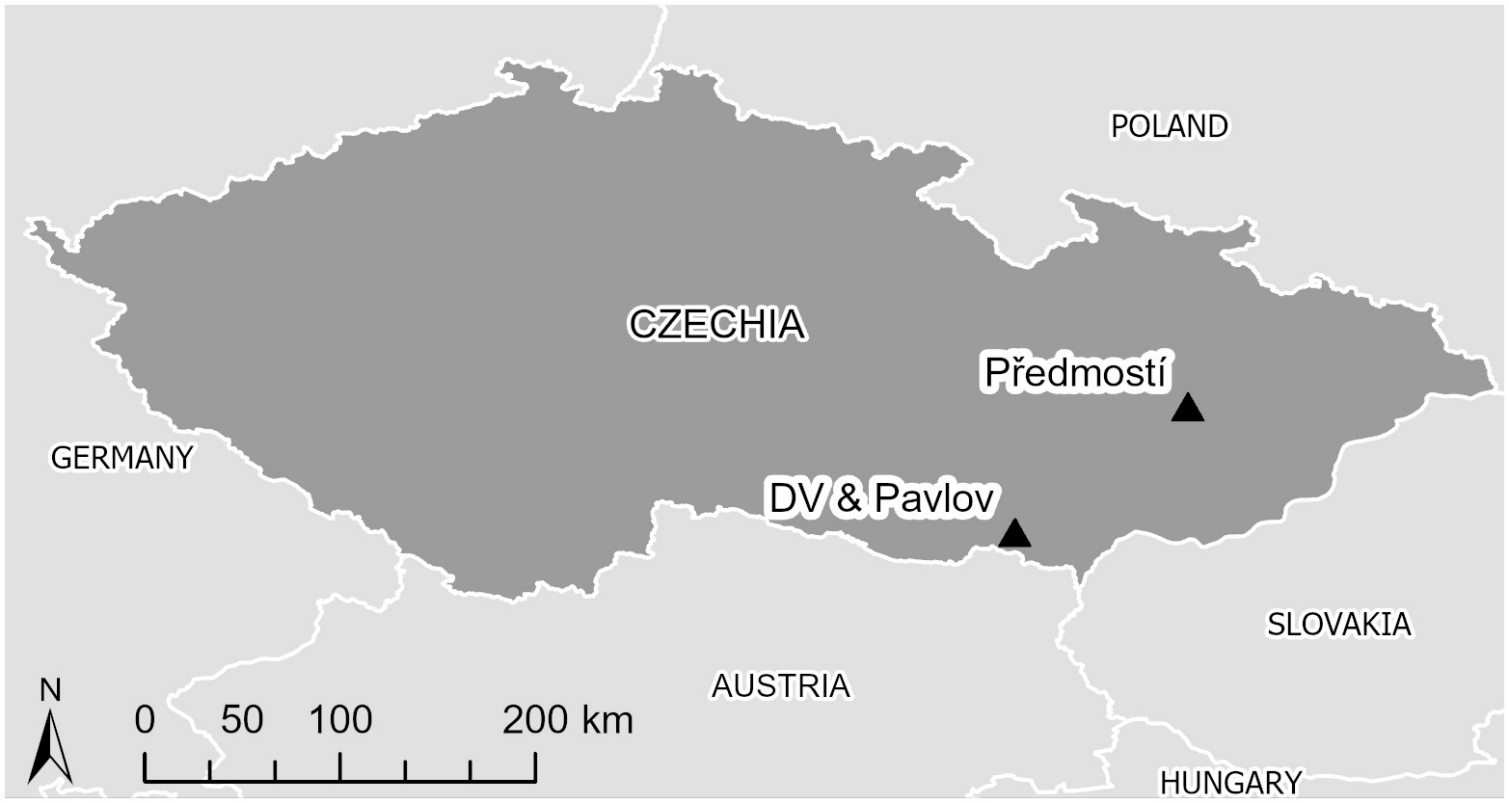
Map of Czechia showing location of sites mentioned in paper. Data used in this study were collected from five Pavlovian sites in southern Czechia: Dolní Věstonice I and II (DV I and II), Pavlov I and VI, and Předmostí. Map data: Sevdari, Kristian; Marmullaku, Drin (2023). Shapefile of European countries. Technical University of Denmark. Dataset. https://doi.org/10.11583/DTU.23686383. This dataset is distributed under a CCBY-NC-SA 4.0 license.

Data used in this study were collected from artifacts excavated from DV I, DV II, Pavlov I, Pavlov VI, and Předmostí, five archaeological sites in Czechia (Fig 1). Calibrated radiocarbon dates on human bones from burials at Pavlov I and DV II range between 31,270 calBP and 29,260 calBP [53; see also 54, 55; for comparable dates]. Collectively, these sites form part of the Pavlovian cultural complex that existed in the region of Moravia, northern Austria and southern Poland. A regional variant of the Gravettian, the Pavlovian is defined by its large open-air aggregation sites which preserve rich stone, bone, and antler material culture, both functional and decorative, complex symbolic burials, and lithic assemblages characterized by large numbers of blades, bladelets and burins often made by exotic raw materials [56, 57].

This regional technocomplex is differentiated from other European Gravettian assemblages by the presence of the ceramic artifacts that are the focus of this paper. No ceramic pottery or functional vessels have been found in the European Upper Paleolithic. Instead, more than 12,000 ceramic figurines, figurine fragments/elements, and non-figurative pellets have been recovered from these sites with the two largest assemblages coming from DV I and Pavlov I. These artifacts are some of the oldest ceramic objects in the world (see [58] for a discussion of Paleolithic ceramic artifacts that postdate the Last Glacial Maximum).

### Sample

The sample used in this study consists of high resolution photographs of 489 ceramic artifacts from DV I, DV II [54], Pavlov I, Pavlov VI [59, 60], and Předmostí. The collection includes figurines of animals (both carnivores and herbivores), male and female human forms, figurine fragments, non-diagnostic fragments that may have been figurine fragments but are no longer identifiable as such, and pellets that may have been made during experimentation with the novel material (Table 1). Pellets were identified as being whole artifacts (no visible breaks), but non-diagnostic in form and lacking any evidence of figurative representation. Following convention, ‘clay’ is used in this study to denote a raw material, specifically a material that is composed mainly of fine particles of hydrous aluminum silicates and other minerals; ‘ceramic’ is defined as a synthetic material composed of sedimentary material that is often (but not always) clay rich, which is then fired; and ‘pottery’ refers to ceramics that are formed to make functional vessels. Accordingly, ‘ceramics’ is used to refer to our sample because Upper Paleolithic peoples did not apply fired clay technology to making pottery [61]. One reason for this may be “that rather than risk a shift to the development of a novel ceramic vessel technology…people preferred to continue with the well-established tradition of making baskets and plant fibre containers, the evidence for which was found in favorable preservation conditions at sites such as Dolní Věstonice and Pavlov" [61, p.49 and references therein].

**Table 1:** Ceramic artifacts included in this study.

Site	Anthropomorphic	Zoomorphic	Undiagnostic	Pellets	Total
DV I	27	51	140	0	218
DV II	0	0	19	0	19
Pavlov I	22	41	45	132	240
Pavlov VI	0	3	8	0	11
Předmostí	0	1	0	0	1
**Total**	49	96	212	132	489

Ceramic artifacts included in this study from the major Pavlovian sites with ceramic artifacts from south-east Czechia. This sample includes all the artifacts that are identifiably representational, and a smaller sub-assemblage of the so-called "pellets" from Pavlov I.

We compare these ceramics to ivory, bone, antler, and soft stone (schist/shale) artifacts excavated from DV I and Pavlov I, which include both figurative and non-figurative art and ornamentation. At Pavlov I, this includes 87 ivory, 15 bone, and 9 antler artifacts. At DV I, a smaller assemblage of 28 ivory artifacts and 1 bone artifact were available to study. Organic and soft stone symbolic material culture long predates the Pavlovian [see 62, 63] and symbolic artifacts in these raw materials have been found in many distinct regional techno-complexes, throughout the Upper Paleolithic.

### Data collection and analysis

Digital photographs were taken of all artifacts discussed in this paper, as well as microscopic photographs (up to 40x magnification) of a smaller sub-sample of the assemblage. All artifacts were measured in three dimensions using digital calipers (in mm), and were weighed in grams using digital scales. All measurements were entered into an Excel spreadsheet. Other quantitative and qualitative data about the artifacts were also collected and recorded in the spreadsheets (e.g., subject matter, color, surface hardness, presence/absence of superficial engravings, state of preservation). Ceramic and ivory artifacts were compared in terms of size, asymmetry, evidence of experimentation, complexity of technique and chaîne opératoire and productivity. Both asymmetry and evidence of experimentation were recorded as binary criteria: if there was any evidence of experimentation during production, or if the figurine was asymmetrical in terms of its appearance/style/form, these characteristics were recorded as present in the artifact. Some figurines preserved more than one form of evidence of children or novices, whereas others preserved just one strong signature characteristic (see Results). Ceramics were also compared to bone and antler artifacts in relation to all of these variables except for degree of asymmetry and experimentation as bone and antler artifacts are non-figurative, rendering these variables less directly applicable. Pavlovian craftspeople carved and engraved geometric and decorative patterns onto the surface of the bones of various herbivores and carnivores, and onto the surface of reindeer antler, but, on current evidence, they never sculpted these materials to make representational depictions of humans or animals [64].

## Results

### Size

**Fig 2:**
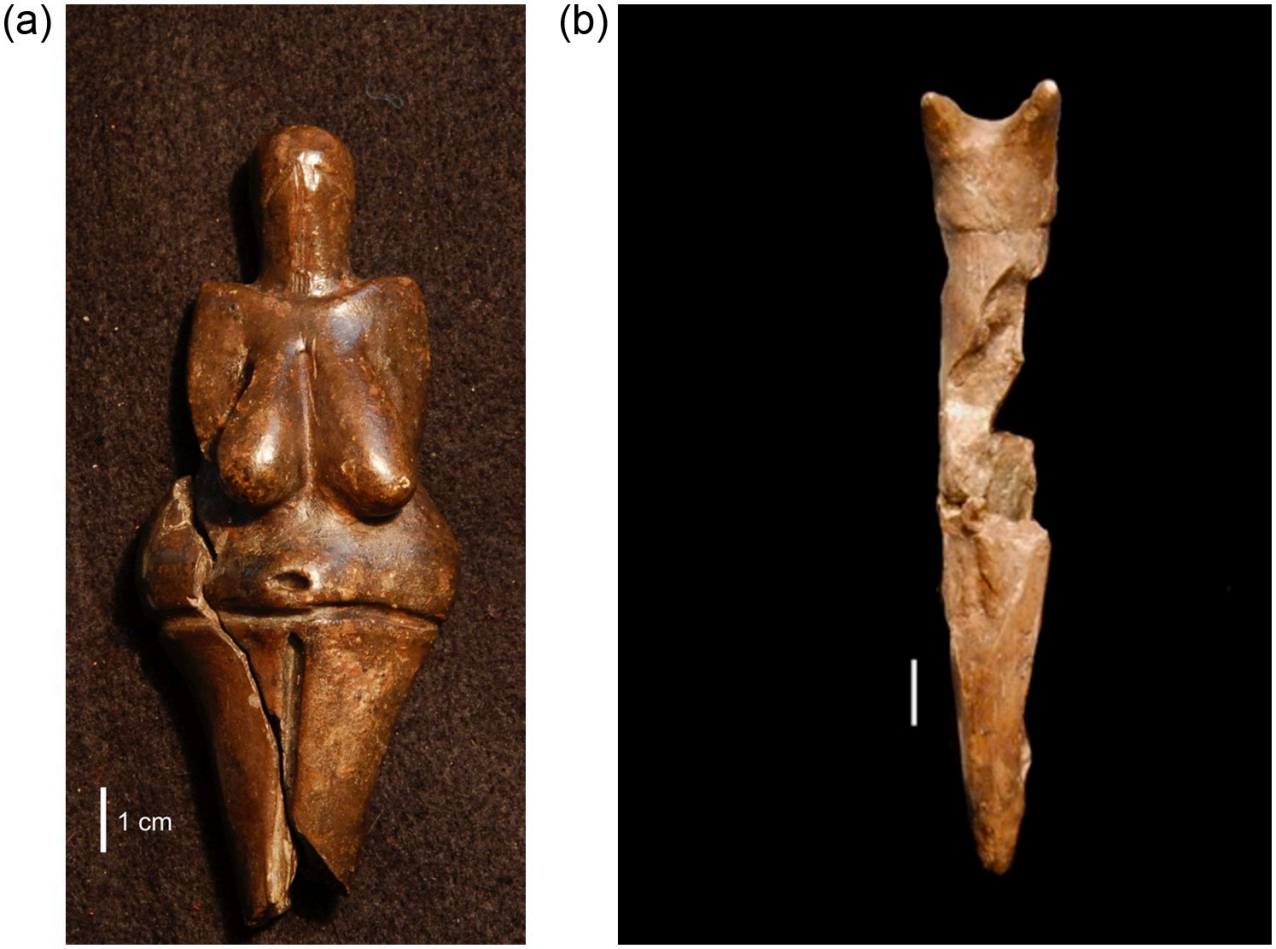
Larger ceramic figurines from DV 1. (a) Female figurine from DV I dating to ca. 27,000 BP is 111 mm in height and 43 mm in maximum width. It was created from a loess based clay fired at a relatively low temperature (500–800 °C) [67]. (Photo: Rebecca Farbstein, taken with permission from Martin Oliva at the Moravian Museum, Anthropos Institute); (b) stylized ‘owl’ figurine, which depicts characteristic ear ‘tufts’ and an abstracted and elongated body (118 mm height, 21.4 mm in maximum width) (Photo: Rebecca Farbstein, taken with permission from Martin Oliva at the Moravian Museum, Brno).

Compared to other materials used to make portable art such as ivory, bone and antler, ceramics are significantly smaller (Tables 2 and 3). At both Pavlov I and DV I, ceramic anthropomorphic and zoomorphic figurines are half the size of similar artifacts made of ivory while non-figurative/non-diagnostic pieces are half the size of non-figurative ivory artifacts, and even smaller still than non-figurative pieces made of bone or antler. The only exceptions to this pattern are the finely made “masterpieces” from these sites such as the virtually complete female figurine from DV I (Fig 2a). This figurine depicts a woman with abstracted facial features, large breasts, a deeply engraved navel and waistline or waistband. Only her lower legs and feet are missing. The break suggests that at least some portion of the lower limbs was originally depicted, although it is impossible to determine if feet were modeled. Another example of an unusually large ceramic figurine is a depiction of an owl from DV I (artifact # DV 28) ([65] for discussion of the multilayered relationship between owls and humans in the Pavlovian). This figurine’s head is damaged but retains the characteristic ear “tufts” associated with owls from DV I (Fig 2b). A third unusually large ceramic object is the damaged and fragmented but refitted mammoth figurine from Pavlov I [66].

**Table 2:** Size comparisons at Pavlov 1 (mm).

	Ceramic	Ivory	Bone	Antler
Maximum Artifact Size	61.3	370	132	220
Minimum Artifact Size	8.3	9.5	35	61
Average Size	19.3	70.9	90.5	151.8

Comparison of maximum, minimum and average sizes (mm) of diagnostically figurative, non-diagnostic, and ornamental artifacts made from ceramic, ivory, bone and schist/shale at DV I.

**Table 3:** Size comparisons at VD 1 (mm).

	Ceramic	Ivory	Bone*	Schist/shale
Maximum Artifact Size	118.7	395	173	111.5
Minimum Artifact Size	6.4	5.5	173	22
Average Size	23	37.5	173	53.3

Comparison of maximum, minimum and average sizes (mm) of diagnostically figurative, non-diagnostic, and ornamental artifacts made from ceramic, ivory, bone and schist/shale at DV I.

*There is only one ornamental bone artifact from DV I: a wolf radius engraved with a series of parallel hatch marks

**Fig 3:**
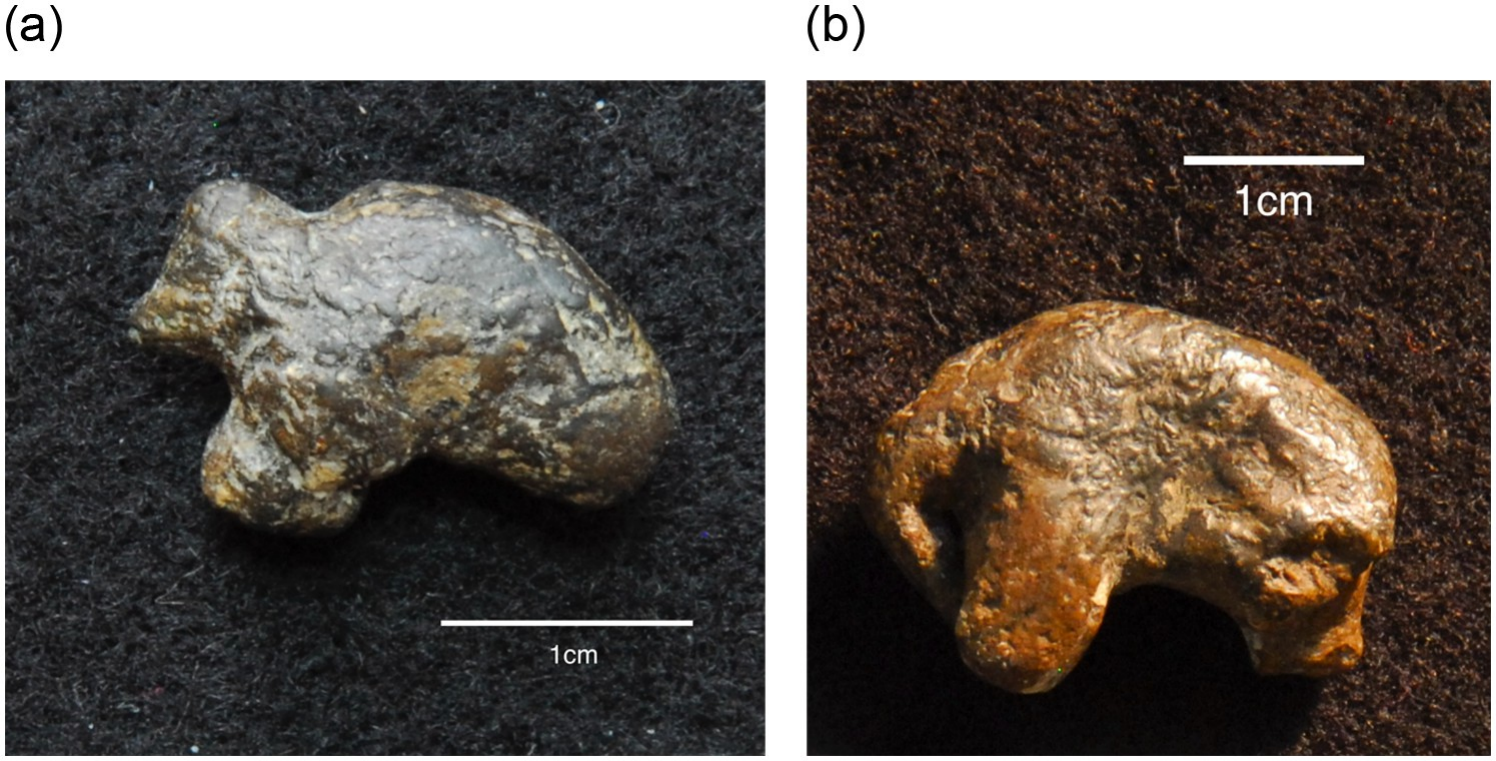
Examples of smaller ceramic figurines. (a) Small ceramic mammoth from Pavlov I, measuring 14.4 x 21.8 x 9.5 mm (Photo: Rebecca Farbstein, taken with permission from J. Svoboda at the Institute of Archaeology, Dolní Věstonice). (b) Another small ceramic mammoth from the neighboring site of DV I, measuring 21.3 x 30.6 x 13.3 mm (Photo: Rebecca Farbstein, taken with permission from M. Oliva at the Moravian Museum, Anthropos Institute). Both illustrate the type of craftsmanship typical of very small ceramic figurines from these sites.

All three of these examples are noticeably larger than the vast majority of other Pavlovian ceramic figurines, which are by-and-large smaller in size and less finely made. For instance, a tiny ceramic mammoth from Pavlov I illustrates the full size range at the site, as this complete figurine measures only 14.4 x 21.8 x 9.5 mm in overall dimensions (Fig 3a). Although the characteristic silhouette of a mammoth is unmistakable, this figurine is otherwise a simplified and generalized depiction of this animal, rather than a detailed and realistic masterpiece. A similarly small ceramic mammoth from DV I (21.3 x 30.6 x 13.3 mm) supports the notion that very small ceramic figurines often exhibited less detailed or skillful finishes (Fig 3b). Another very small zoomorphic figurine from the same site measures just 11.3 x 17.6 x 5.6 mm. The subject matter is unclear, perhaps because of both the small size of the figurine and its abstracted style of depiction. These artifacts suggest there may be a relationship between the largest figurines and more skilled craftsmanship, and the smaller ones and somewhat less skillful production, perhaps coupled with more experimentation.

### Asymmetry

**Fig 4:**
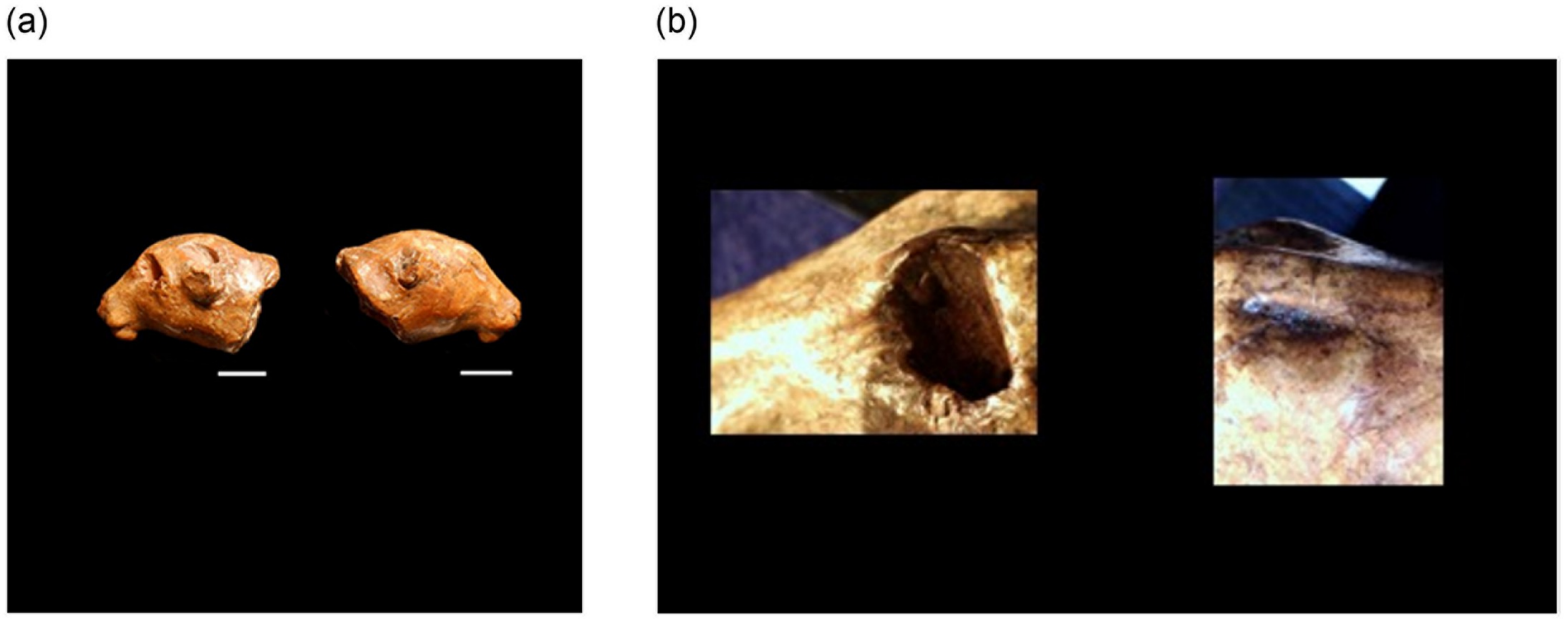
Macroscopic and microscopic photos of ceramic lion’s head from DV1. (a) macroscopic photos of the two sides of a single ceramic lion’s head from DV I; (b) microscopic photos (40X) of the eyes of this figurine, showing how the craftsperson used two different techniques to create the eyes. The degree of experimentation and the lack of attention to symmetry in a ceramic lion’s head from DV I suggests the artifact may be the product of a novice maker. Length = 45 mm. Height/Depth = 25 mm (Photos: Rebecca Farbstein).

The next variable considered was degree of asymmetry. At both Pavlov I and DV I, ceramics were found to be less symmetrical than portable art made from ivory (see Table 4). For example, there are assemblages of ceramic animal heads found at both Pavlov I and DV I. Some of them demonstrate that the maker created the face in one way on one side and then used a different technique to make the other side of the face to produce a completely different result. For example, on one side of a purported lion’s head (Fig 4a and 4b), found at DV I, the eye is a slit, a simple engraving across the surface. On the other side, the eye is a perforation that penetrates all the way through the head. Each one of the lion’s ears is rendered completely differently as well. The degree of experimentation evident even in the lion’s head suggests that symmetry of its final form was not a priority for this object; more broadly, the rest of the ceramics assemblage confirms that representational asymmetry was common in Pavlovian ceramics, but totally absent when working with ivory at the same sites.

**Table 4:** A comparison of the percentage of artifacts from DV 1 and Pavlov 1 exhibiting representational asymmetry, experimentation and seriation by material type.

	Ceramic at DV I	Ivory at DV I	Ceramic at Pavlov I	Ivory at Pavlov I
Percentage of Figurative Assemblage Displaying Representational Asymmetry	17%	0%	42%	0%
Percentage of Assemblage demonstrating experimentation/heterogeneity	58%	40%	49%	28%
Percentage of Assemblage demonstrating seriation/homogeneity	42%	60%	51%	72%

### Experimentation vs. seriation

**Fig 5:**
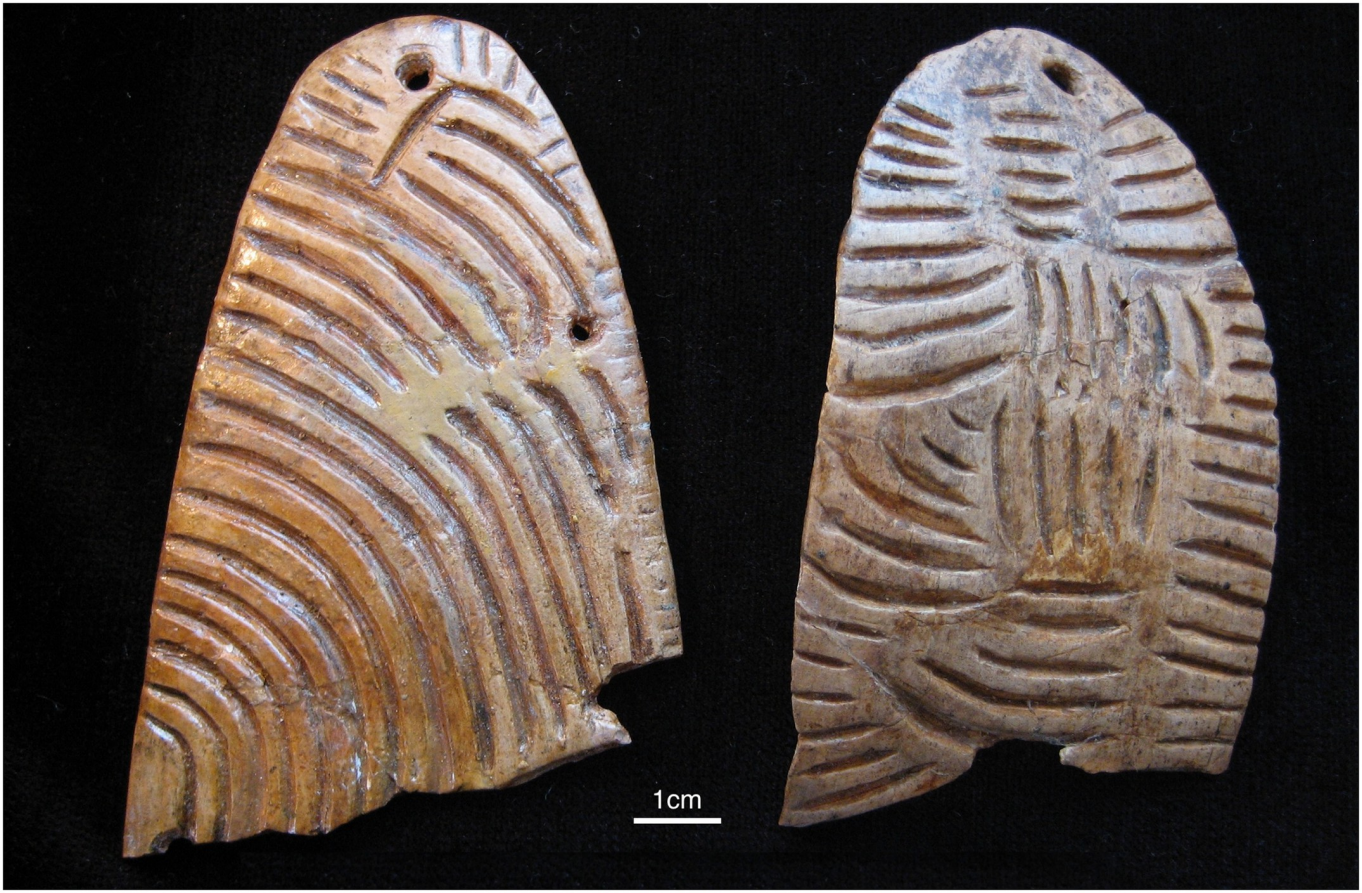
Two of the engraved ivory diadems from Pavlov I. Both of these fragments are more than 90 mm in length. Full diadems from the same site measure up to 175 mm in length and approximately 35 mm in width. All diadems were made from ivory lamellae and thus have a relatively uniform depth of c. 2.5–3 mm (Photo: Rebecca Farbstein).

**Fig 6:**
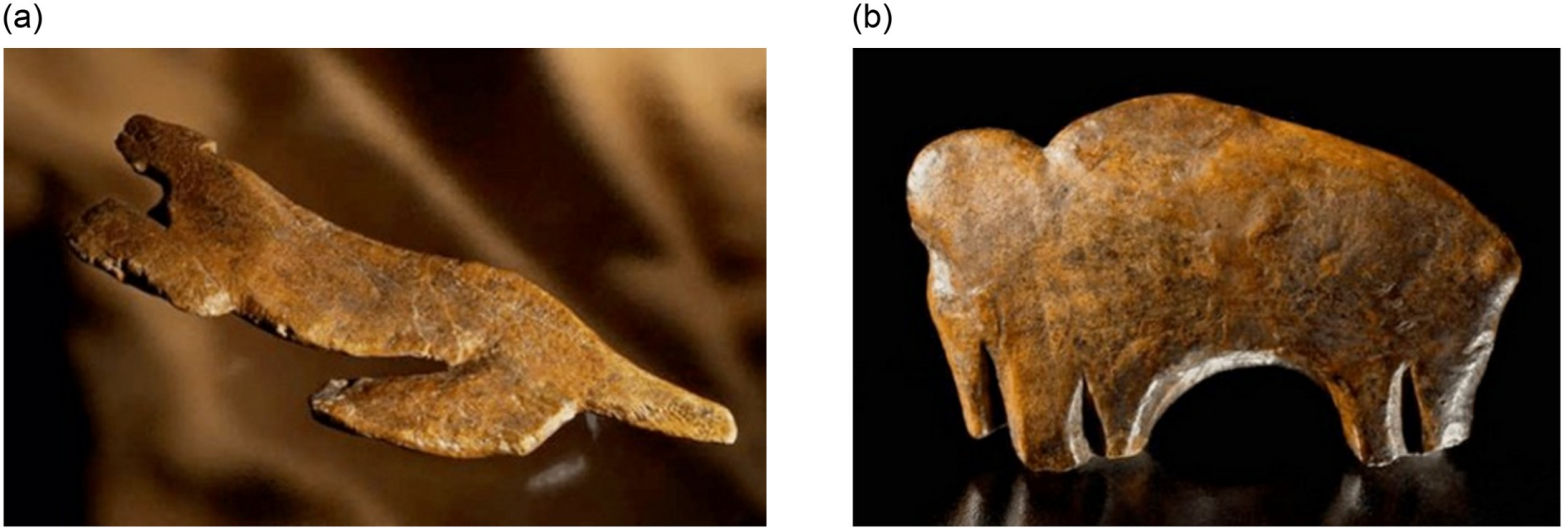
Ivory lion and mammoth from Pavlov 1. (a) Lion (length = 215 mm) and (b) mammoth (39 x 69 x 10 mm) from Pavlov I. Both were made from ivory lamellae and demonstrate the strong socio-technical preference for silhouetted representations of animals at this archaeological site (Photos: Courtesy of Martin Frouz and Martin Novák and the Institute of Archaeology of the Czech Academy of Sciences, Brno; permission granted by Martin Novák to publish under a CC BY 4.0 license).

**Fig 7:**
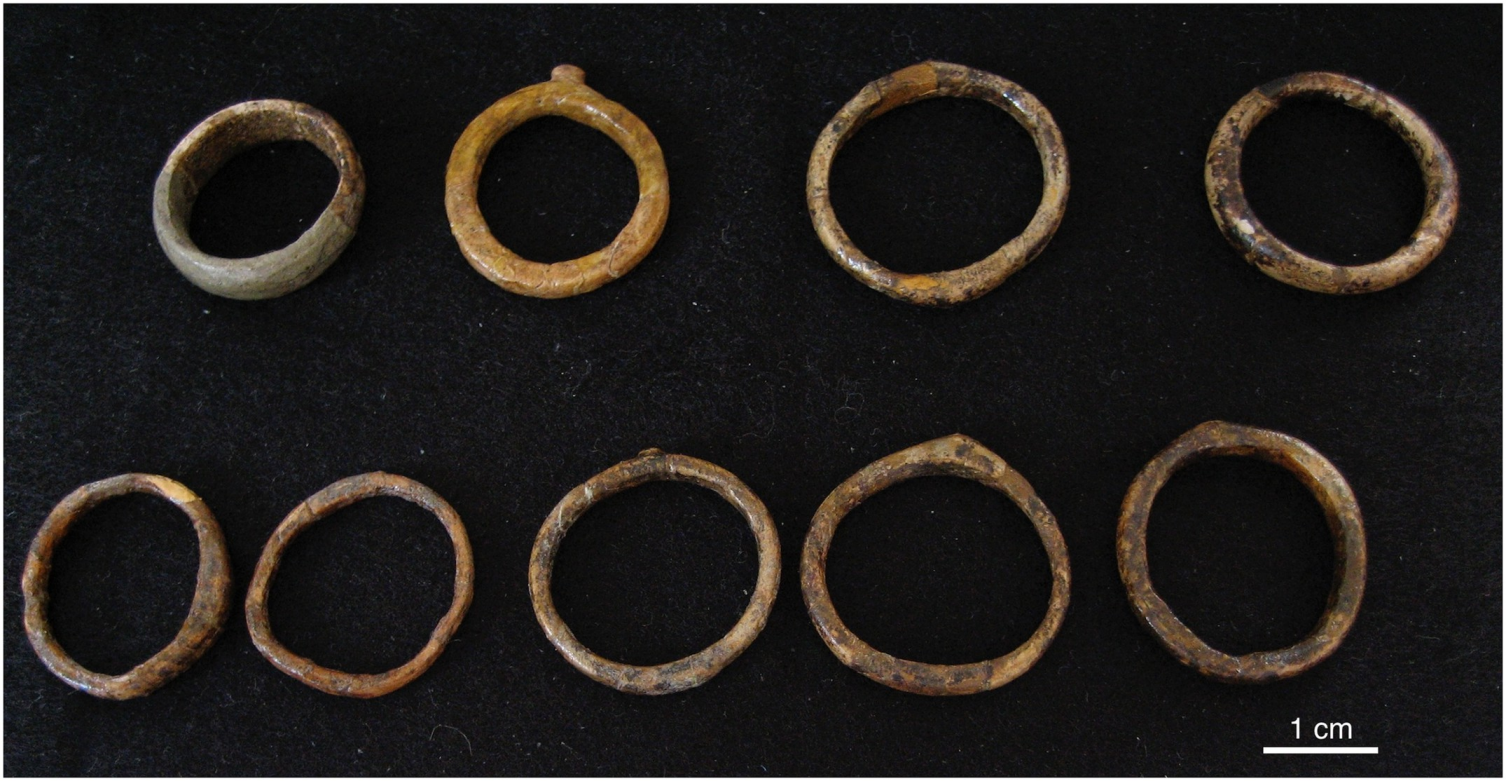
Series of ivory rings from Pavlov I. These ivory rings demonstrate a high degree of technological and stylistic homogeneity. Diameter ranges between 23–31.5 mm. Width of the rings ranges between 2.5–7.5 mm (Photo: Rebecca Farbstein).

The significance of the degree of experimentation apparent in the ceramics becomes even more striking when they are compared to ivory art at the same sites. For example, both figurative and non-figurative ivory art from Pavlov I displays a higher degree of seriation and a lower degree of experimentation than the ceramic artifacts from the same site (Table 4). At this site there are a large number of highly standardized objects described as diadems that may have been worn as headbands or bracelets (Fig 5). They were all manufactured in the same way and exhibit broadly the same finished form [64, 68, 69]. Figurative art depicting a range of subjects, including lions, mammoths, and a purported ‘owl’, also often echoed this techno-stylistic approach to creating flattened silhouettes (Fig 6a and 6b). A series of ivory rings from Pavlov I reinforces this socio-technical pattern; when working with organic materials, Pavlovian craftspeople often privileged stylistic and technological homogeneity and seriation (Fig 7).

**Fig 8:**
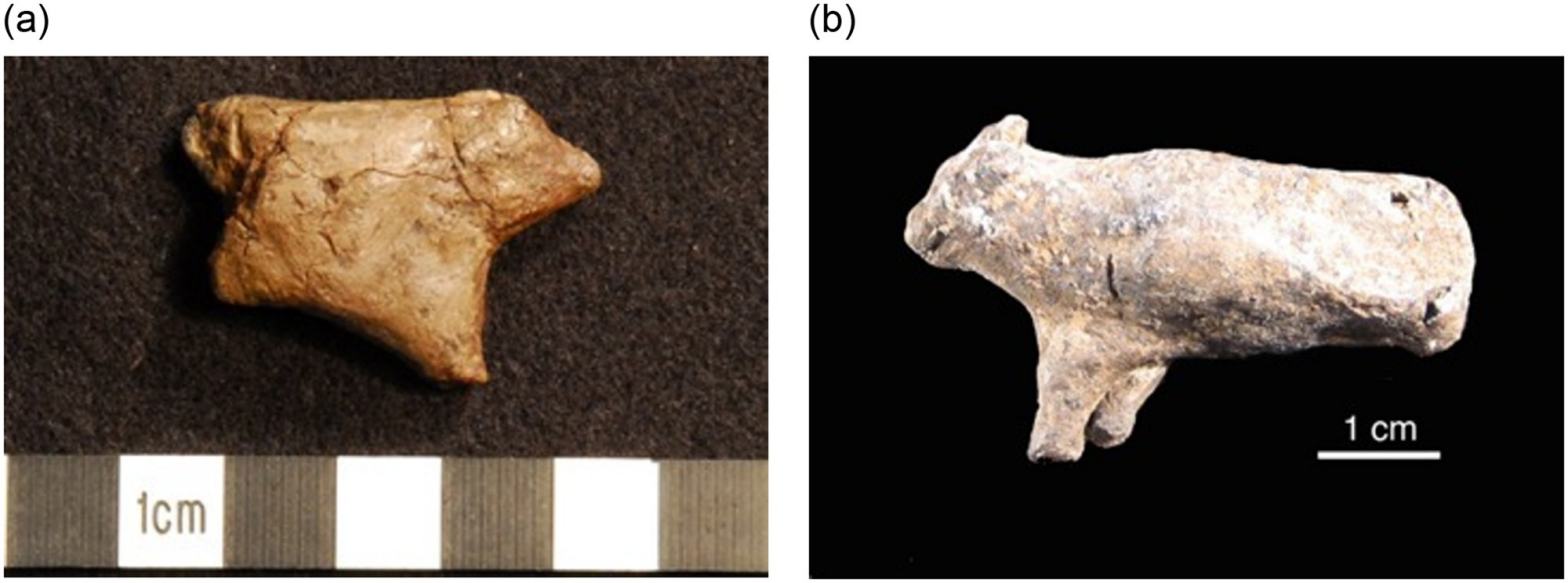
Zoomorphic ceramic figurines from DV 1. (a) Zoomorphic ceramic figurine from DV I (DV 44), measuring 3.2 cm tall, 4.7 cm long. Forelimbs have been formed as a single appendage subsequently attached to the torso of the animal; (b) Zoomorphic figurine (sometimes interpreted as a bovid) from DV I (artifact # DV 33), measuring 2.5 cm tall, 4.8 cm long. The animal’s forelimbs were rolled separately, and subsequently joined both to each other, and to the torso of the figurine which was made separately (Photos: Rebecca Farbstein).

By contrast the ceramic figures evince much more heterogeneity. They are more diverse both in terms of the technique, subject and the finished form/finished aesthetics. For example, sometimes an animal is made with its forelimbs as two separate limbs while at other times makers created a more generalized form, rendering the forelimbs as one (Fig 8a and 8b). At least 30 ceramic leg fragments were excavated from Pavlov I which illustrate how craftspeople experimented with the range of ways both human and animal legs might be rendered in this medium. Sometimes a realistic foot was modeled. In other instances the base of the leg was flattened, perhaps to enable the artifact to stand upright, but a realistic foot was not modeled. On other artifacts, there is no evidence that an iconographic foot or flattened surface was modeled; instead, the leg was rolled to a tapered extremity. Greater variation is also noticeable in the use of surface engraving, decoration, and ornamentation across the assemblage, and even in the production of a single artifact.

### Technique/chaîne opératoire

**Fig 9:**
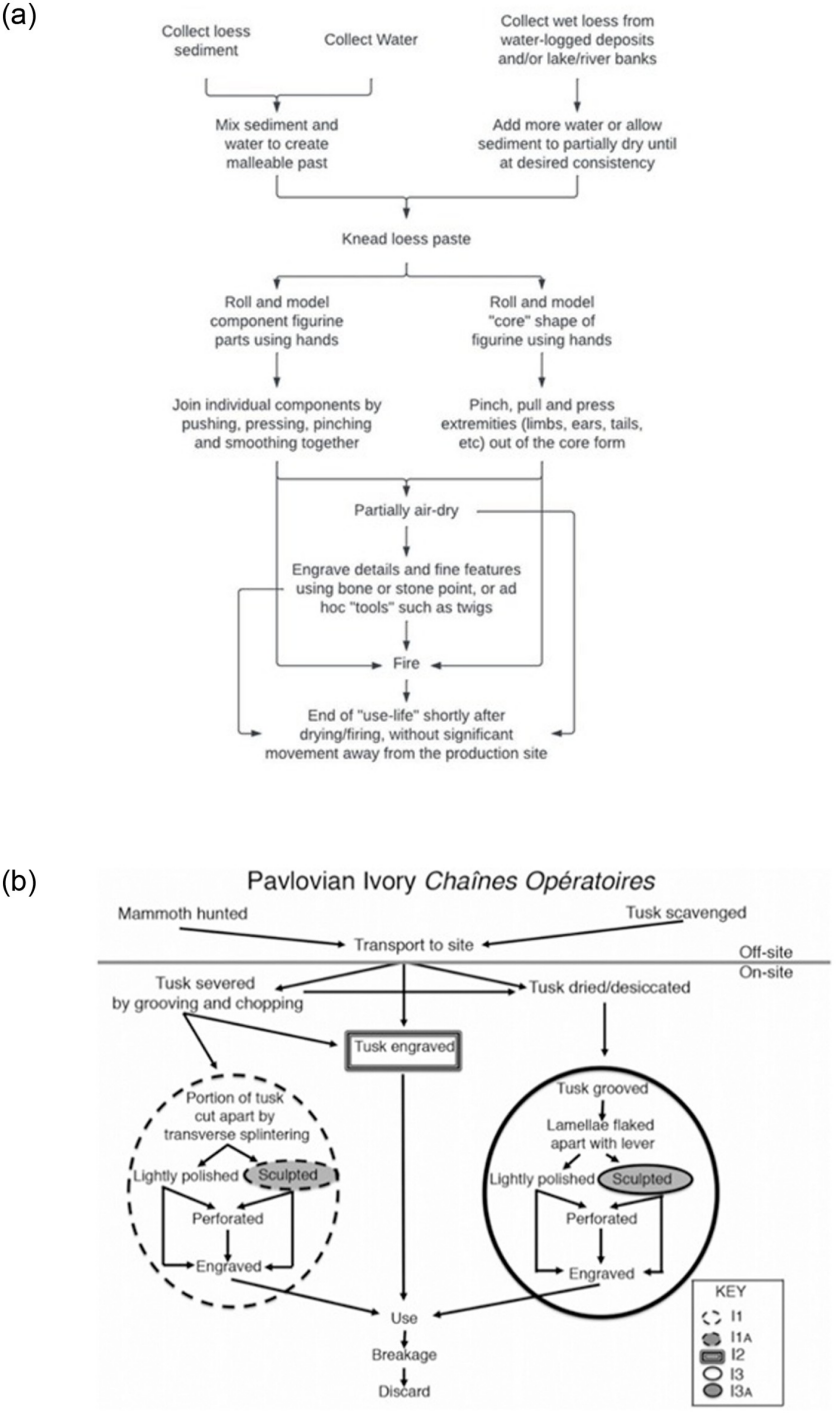
Comparative chaînes opératoires of ceramic and ivory production. (a) Diagram of the Pavlovian ceramic chaînes opératoires, illustrating five distinct variations in the production sequence for figurines made in this material; (b) Comparative diagram of Pavlovian ivory chaînes opératoires, illustrating six distinct variations in the production sequence, the use of a variety of synthetic tools during production, and evidence of a longer temporal investment during production of art in this raw material.

Pavlovian craftspeople employed a range of different *chaînes opératoires* when making ceramic artifacts, although all of them shared the same initial stages of production (Fig 9a). The local loess-based sediment was collected from the immediate landscape, either in a slightly wet form or dry, in which case it would have been mixed with water to form a paste. This paste was then shaped by hand into various forms. Often component parts of a figurine, for instance, its torso, head, arms and legs, were formed separately, and subsequently joined together, in a technique described as ‘additive’ [66, 67, 70–74]. However, a significant proportion of the figurines were made using pinching, pressing, and pulling actions to create a figurine from a core shape, without additive technology [66]. Some figurines were decorated with surface engravings, made with a range of tools that might have included both stone and bone tools, as well as fingernails. Other artifacts were not decorated with engravings or carvings.

Relative to ceramics, ivory, bone and antler are temporally elaborated materials. In other words, it takes a significantly longer amount of time to collect, process and make portable art objects from them (Fig 9b). For example, to obtain ivory, one would need to hunt a mammoth or scavenge a tusk. This task would necessitate leaving the site itself and coordinating a group of people. This task further requires a degree of skillfulness, likely acquired through observing and/or participating in past scavenging or hunting missions and listening to stories of elders (see, for example, [75]). Furthermore, a variety of specialized tools are needed to process these materials through techniques such as scraping, splintering, sculpting, and engraving. Bone and antler, which were primarily used to make non-figurative art at Pavlovian sites, necessitated similarly time- and labor-intense manufacturing techniques that required social collaboration, the use of specialized skills throughout the chaîne opératoire, and the use of a variety of specialized manufactured tools.

Another important distinction between ceramics and the other materials used to make Pavlovian art is that ceramics are ‘synthetic’; they require the use of fire to transform the ‘raw’ sedimentary paste into the final, durable ceramic form. This transformation of the material itself is an entirely new process associated exclusively with ceramics, which stands in stark contrast to the established ivory, bone, and antler technologies.

### Non-productivity

Pavlovian sites are known not only for their ceramics but for their burials as well [76–79]. In some of these burials, highly diverse assemblages of ornamental culture were interred with the bodies. For example, at Brno II, a 23,680 year old Pavlovian site roughly 40 km north of the DV/Pavlov site cluster, archaeologists recovered the remains of an adult male [80, 81]. Associated with the man was a figurine depicting a man, known as the ‘marionette figure’ created from 3 interlocking pieces of ivory. Alongside the figurine were 13 disks made of mammoth molars and a variety of soft stones [82]. In other burials, such as the famous Triple Burial from DV II, perforated animal teeth and shells were included in the burials [76, 77, 83]. What is most remarkable is that no ceramics have been discovered in a Pavlovian burial. Given the abundance of ceramic artifacts found dispersed across the occupation sites, and the diversity of materials found within the burials, we argue that ceramic objects were purposely excluded from the interments.

By contrast, ceramics are found throughout the occupation sites, with some noteworthy concentrations of these artifacts in or immediately next to hearth structures. They were made adjacent to the fire and then placed in the fire as part of their production sequence, (chaîne opératoire) and they remained there. For example, at DV I, ceramic figurines were discovered inside and immediately around the periphery of the hearth. Furthermore, Farbstein and Davies [84] note that some figurines are so soft that it is clear that they were not fired but rather left to air dry. From these observations, it is clear that the ceramics are not moving very far from where they are made. This is a very different life history than what is documented for other symbolic culture at these sites. Not only is it shorter but there is an element of non-productivity with the ceramic figurines. They were not being manufactured for a specific purpose and this distinguishes them from the other material culture made from the other raw materials at the sites.

## Discussion

### Children as novice makers

Returning to the research questions posed at the beginning of this paper—can the ceramic products of novices be distinguished from those made by experts? If so, can we tell if these novices were children? First, the data presented in this paper allow us to distinguish between two different kinds of craftspeople and the types of artifacts they made. Ceramics are smaller, more asymmetrical, and the result of simpler, shorter production sequences and techniques than artifacts made from other materials. Furthermore, they demonstrate a higher degree of experimentation, techno-stylistic heterogeneity, and non-productivity. In this instance, at these sites, ceramics seem to be the type of material culture made by novices.

Second, there are multiple lines of evidence supporting the hypothesis that in many cases these novices were children. Not only are the variables considered in this study typical of child learners but some of the Pavlovian ceramics preserve the fingerprints of the people who made them and the majority of these belong to children. Children’s fingerprints can be distinguished from adult fingerprints by (1) overall size, (2) difference in breadth of epidermal ridges, and (3) ridge proportions [24]. For example, archaeologists studying a fingerprint on the back of the DV I figurine discussed above estimate that it belongs to a subadult of 11–13 years [36]. Similarly, a study of 2635 fired worked and unworked clay objects from Pavlov 1 uncovered 48 imprints on 29 objects [37; see also 85]. Using as a comparison a range of epidermal ridge breadths derived from a modern Czech sample, these researchers determined that these imprints most closely matched children between the ages of 6–10 and adolescents between the ages of 10–15. The mean epidermal ridge breadth of imprints on eight objects with the best preserved traces was 0.383 mm corresponding to an individual of 11.8 years old. At a minimum, this evidence documents the presence of children during the production process and thus their integration into a community of practice around ceramics. However, as Králík and Novotný [85, p. 473] note, “in most of the investigated Pavlovian objects, the imprint is also a trace connected with the molding process, so the author of the print is also likely to be the maker of the artifact. This is especially relevant in the last phases of molding.”

In combination with the evidence for novices, the fingerprint data strongly suggest that children made at least some of the Pavlovian ceramics. Comparable archaeological evidence for children’s participation in ceramic production in other time periods include fingerprints on artifacts such as seven miniature ceramic vessels from Těšetice-Kyjovice, a Neolithic site in Czechia [37], animal figurines in Egypt dating to 3,800–3,7000 BP [86], pottery shards from six Early and Middle Bronze Age sites in England [87], a variety of clay objects from Early and Late Bronze Age sites in Israel and Syria [43, 88–91], Iron Age pots in Spain [92], 3400 year-old clay tablets from Knossos [93], pottery from a late 14th century English site [94], 57 Sinagua figurines in Arizona dating to 1425 CE [26, 95, 96] and ‘learner vessels’ from a Late Woodland Iroquoian village in Canada [49, 50].

Kamp [96] notes that child novices make errors in tempering, drying and firing; and they may not follow the accepted procedures, skipping a step or producing a different shape. This may be because they do not completely understand what the final product should look like. Králík and colleagues [37; see also 36] observe that many figurines and figurine fragments exhibit cracks typical of thermal shock indicating that ceramics were often fired before they were sufficiently dry. Vandiver et al. [67] argue that fragments of ceramic figurines found in association with hearth structures at Pavlovian sites in Czechia suggest they were ritually exploded in what they term a pyrotechnical performance [70]. They assert that the properties of the loess paste make accidental thermal shock “improbable” and instead they believe that it required “intentional effort and practice” to explode these figurines into small fragments. While this scenario is a possibility, it is also possible that this phenomena is further evidence of novices [8, 32]. The ceramic human and animal figures from Pavlovian sites are solid pieces. Even when fired at very low temperatures, it is quite easy for solid pieces, relative to hollow ones, to explode if too wet. Even after a couple of days of drying, solid ceramic objects can appear dry on the outside but remain wet on the inside [97].

Children often enter communities of practice centered on ceramics through playing with clay and by making simple animal figures and small pots to use as toys or to practice cooking [e.g., 24, 35, 47, 48, 98–100]. In fact, Lew-Levy et al. [101] and Riede et al. [102] demonstrate the near universality of the association between children and these types of toys. According to Kamp [24], animal figurines often exhibit variability in execution, fingernail and fingerprint marks, lack detail, and are only rarely smoothed. Furthermore, the 2 sides are sometimes asymmetrical or out of proportion and heads or limbs are often missing because they were not securely attached. All of these features are observable in our sample of Pavlovian animal figurines [see also 84]. Králík and Novotný [85] suggest a scenario where Paleolithic children might have played at hunting with these animal figures, damaging them in the process; playing with the figures would have allowed children “to learn and explore vital aspects of ecologically and hence adaptively relevant knowledge about animal behavior” [12, p.54].

### Implications for socialization and learning through play

Cross-culturally, children, most often girls, begin playing with clay by two to five years of age [24, 92, 99, 103–109 but see also 100] and through play they learn the properties, or affordances, of the material [8]. Child-sized finger holes in clay at Upper Paleolithic sites suggest this behavior may have a great antiquity [e.g., 110]. They learn the steps that must be followed to successfully produce a ceramic artifact even though they may master some tasks earlier than others [40]. This learning is often integrated into other everyday tasks. For example, in an ethnographic study among the Kusasi of Ghana, children begin using clay between the ages of five to seven years and transition into a “proper learning of pottery production” when they reach eleven or twelve years old [111, p.93]. The Kusasi do not have a specific word for ‘apprentice’, which Trias et al. [111, p. 93] argue is evidence of “a fluid and multiform concept of pottery learning closely interrelated to the rest of the roles and activities taking place in the domestic sphere.” Children often assist in the pottery making process by collecting and preparing raw materials, e.g., bringing in clay, temper and water, tempering the clay (i.e., adding non-plastic material to clay to prevent shrinkage and cracking during the firing process), possibly grinding the clay and temper, removing foreign particles by hand, kneading the clay and collecting firewood to fire the clay [5, 24, 40, 112]. They may aid in clean-up and performing less technical aspects of the process until they develop both the muscle memory and cognitive capacity necessary to undertake all stages in the production process. In this way, ceramics can be thought of forming part of a ‘taskscape’. Ingold ([113], p. 158) writes, “every task takes its meaning from its position within an ensemble of tasks, performed in parallel, and usually by many people working together.” Thus ceramics may be part of a larger ensemble of tasks even though the task of collecting materials for the production of ceramics themselves is quite simple. At the French site Tuc D’Audoubert, known for its molded (unfired) clay bison, archaeologists working with professional trackers identified several sets of tracks left by an adult male and an adolescent boy as they twice made their way to and from a clay pit within the cave [114]. The depth of the tracks exiting the pit suggest the pair were carrying armloads of clay with them—perhaps as much as a total of 45 kg over the two trips based on the amount of clay extracted.

Children exhibit agency in their learning. As they get older they increasingly play a role in choosing what they learn and from whom they learn, including peers and slightly older children by setting goals and initiating interactions [8, 115, 116, see also 117, 118]. Skill is developed through active participation and observation, playful imitation, and experimentation [24, 40] and deliberate practice which involves dividing tasks into chunks, identifying specific goals, focusing on technique and receiving feedback from a teacher or mentor [119]. Accordingly, much of this learning takes place in a social setting. Köhler [40, p.138] uses the term ‘thick experience’ to emphasize the importance of social and spatial proximity in “the transfer of knowledge in making pottery over generations.”

## Conclusion

Since their initial discovery more than a century ago, archaeologists have celebrated Pavlovian ceramics as an exceptional example of one of the earliest iterations of ceramic technology. Decades of research on these artifacts have advanced our understanding of the diversity of Paleolithic art, innovation, and lifeways. Pavlovian ceramics offer insight into the development of novel materials and technologies in the Paleolithic, and into the integration of these technologies into complex hunter-gatherer society. This paper has highlighted that, in comparison to expertly crafted, non-ceramic symbolic material culture that is well-known from this region, Pavlovian ceramics retain several characteristics consistent with production strategies used by novices, including their smaller size, higher degree of asymmetry and experimentation in production, and apparent non-productivity. In addition, the chaînes opératoires for Pavlovian ceramics indicate much more expedience throughout the life history of the artifacts. The local availability of the raw material, combined with the ability to manipulate it without the use of specialized tools, may have contributed to children working with ceramic materials. The fingerprints preserved on the surface of ceramic artifacts, which are consistent with sub-adults, offer direct evidence linking children to the production of Pavlovian ceramic artifacts. One question which remains, and is worth exploring in more detail in future research, is whether, as children grew up, they progressed from more expedient, experimental and exploratory ‘play’ with ceramics to carving, sculpting, and engraving the more time- and labor-intensive ivory, bone, and antler raw materials. It is a tantalizing possibility that the archaeologically recoverable differences in the ceramic and organic Pavlovian art traditions may reflect the difference between ‘art’ made by children and adults. Another important avenue of future research will be to compare ceramics produced during the Pavlovian with Paleolithic ceramics that post-date the Last Glacial Maximum [58] to see if evidence for children as makers is apparent in this period as well.

Much of the last century of research on both portable and parietal Paleolithic art has focused on the most well-preserved and visually striking ‘masterpieces’ [see 120, 121 and references therein]. While this valuable research demonstrated the complex and elaborate symbolic practices of the most skilled members of these societies, shifting the focus to study the production strategies of the broader assemblages, including less stylistically striking artifacts, provides a window into how the broader society, including novices and children, engaged with art-making in the past. The results of our research suggest that it may be possible to uncover children’s work through contextualized, comparative and integrated analyses of certain socially-embedded technologies such as Pavlovian ceramics. One of the most tantalizing interpretations from this research is that Pavlovian children may have been ‘at play’ during their experiments and explorations with ceramic materials. These artifacts may, in fact, be a fortunate and rare materialization of the celebrated, and ephemeral, act of play during childhood.
